# Statistical modeling and optimization of heterogeneous Fenton-like removal of organic pollutant using fibrous catalysts: a full factorial design

**DOI:** 10.1038/s41598-020-72401-z

**Published:** 2020-09-30

**Authors:** Mohammad Neaz Morshed, Md. Nahid Pervez, Nemeshwaree Behary, Nabil Bouazizi, Jinping Guan, Vincent A. Nierstrasz

**Affiliations:** 1 Textile Material Technology, Department of Textile Technology, Faculty of Textiles, Engineering and Business, University of Borås, 50190 Borås, Sweden; 2 Ecole Nationale Supérieure des Arts et Industries Textiles (ENSAIT), GEMTEX Laboratory, 2 allée Louise et Victor Champier BP 30329, 59056 Roubaix, France; 3 Université de Lille, Nord de France, 59000 Lille, France; 4 College of Textile and Clothing Engineering, Soochow University, Suzhou, 215006 China; 5 Swedish Centre for Resource Recovery, Faculty of Textiles, Engineering and Business, University of Borås, 50190 Borås, Sweden

**Keywords:** Heterogeneous catalysis, Pollution remediation

## Abstract

This work focuses on the optimization of heterogeneous Fenton-like removal of organic pollutant (dye) from water using newly developed fibrous catalysts based on a full factorial experimental design. This study aims to approximate the feasibility of heterogeneous Fenton-like removal process and optionally make predictions from this approximation in a form of statistical modeling. The fibrous catalysts were prepared by dispersing zerovalent iron nanoparticles on polyester fabrics (PET) before and after incorporation of either polyamidoamine (PAMAM, –NH_2_) dendrimer, 3-(aminopropyl) triethoxysilane (APTES, –Si–NH_2_) or thioglycerol (SH). The individual effect of two main factors [pH (X1) and concentration of hydrogen peroxide-[H_2_O_2_]_μl_ (X2)] and their interactional effects on the removal process was determined at 95% confidence level by an L^27^ design. The results indicated that increasing the pH over 5 decreases the dye removal efficiency whereas the rise in [H_2_O_2_]_μl_ until equilibrium point increases it. The principal effect of the type of catalysts (PET–NH_2_–Fe, PET–Si–NH_2_–Fe, and PET–SH–Fe) did not show any statistical significance. The factorial experiments demonstrated the existence of a significant synergistic interaction effect between the pH and [H_2_O_2_]_μl_ as expressed by the values of the coefficient of interactions and analysis of variance (ANOVA). Finally, the functionalization of the resultant fibrous catalysts was validated by electrokinetic and X-ray photoelectron spectroscopy analysis. The optimization made from this study are of great importance for rational design and scaling up of fibrous catalyst for green chemistry and environmental applications.

## Introduction

Detoxification of wastewater is a great challenge due to the presence of toxic, complex and diverse organic pollutants that resistant to conventional treatment systems consists of an either-or combination of physical, chemical, and biological processes^1–4^. Advance process such as adsorption, ozone and/or hypochlorite oxidation is effective but undesirable due to their inherent drawback related to cost, efficiency and generation of secondary waste^5–7^.

Recent progress in the removal of toxic pollutants has led to the development of advanced oxidation processes (AOPs). Among them, the oxidation using Fenton's reagent has proved to be a promising and attractive treatment method for its effectiveness towards the destruction of a large number of hazardous and organic pollutants^8,9^. The Fenton process uses iron ions and hydrogen peroxide for the generation of the second most powerful oxidant, i.e., hydroxyl radicals in aqueous solution^10,11^ which is capable of mineralizing organic pollutants in various ways including redox reactions (Eq. 1), dehydrogenation reactions (Eq. 2), electrophilic addition to π systems (Eq. 3) and so on^12,13^.

1 $$^{ \cdot } {\text{OH }} + {\text{ RX}} \to {\text{RX}}^{ + \cdot } + {\text{ OH}}^{ - }$$

2 $${\text{RH}} + {}^{ \cdot }{\text{OH}} \to {}^{ \cdot }{\text{R}} + {\text{H}}_{{2}} {\text{O}}$$

3 $${\text{RHX}} + {}^{ \cdot }{\text{OH}} \to {\text{RHX}}\left( {{\text{OH}}} \right)$$

Ferrous ions consumed during the Fenton reaction are regenerated by further reaction of ferric ions with hydrogen peroxide, as in Eqs. 4–6^14^.

4 $${\text{Fe}}^{{{2} + }} + {\text{H}}_{{2}} {\text{O}}_{{2}} \to {\text{Fe}}^{{{3} + }} + {\text{OH}}^{ - } + {}^{ \cdot }{\text{HO}}$$

5 $${\text{Fe}}^{{{3} + }} + {\text{H}}_{{2}} {\text{O}}_{{2}} \to {\text{Fe}}^{{{2} + }} + {\text{H}}^{ + } + {\text{HO}}_{{2}}^{ \cdot }$$

6 $${\text{Fe}}^{{{3} + }} + {\text{HO}}_{{2}}^{ \cdot } \to {\text{Fe}}^{{{2} + }} + {\text{O}}_{{2}} + {\text{H}}^{ + }$$

Although Fenton and Fenton-like treatments have noteworthy benefits^15–18^, homogeneous Fenton process containing free reagents (iron ions and hydrogen peroxide) have some critical disadvantages including selective reaction conditions (acidic pH), generation of a high amount of iron sludge and single-use reagent which is costly and counterproductive^19,20^. Therefore, much effort has been invested to improve the treatment efficiency of the Fenton reaction process as well as extending their working conditions and cutting down on secondary pollution^21,22^.

Heterogeneous Fenton process using various types of catalysts such as zero-valent iron (ZVI = Fe^0^)^23–26^, goethite (–FeOOH)^27,28^, and Fe_3_O_4_^29–31^, has been extensively studied for pollutant degradation processes. Solid zero-valent iron allows a reduction in iron use, with the potential prospect to work in an operational pH. According to Xu et al.^32^, the zero-valent iron-based Fenton-like system provides better degradability of phenolic compounds even in working pH condition (pH 5–6). However, this process still produces iron sludge (due to the agglomeration of particles and limited recyclability of iron due to easy oxidation) as in homogeneous Fenton process. Hence, researchers introduce immobilization of zerovalent iron particles on solid supports to control the sludge formation and ensure reusability of the catalysts. Our recent studies reported the successful immobilization of zerovalent iron nanoparticles on high porosity support material (fibrous polyester fabric) and explored their effectiveness as a fibrous catalyst toward the removal of various organic and pathogenic contaminants in water through a heterogeneous Fenton process without generating secondary pollutants (iron sludge)^33^.

In general, the Fenton process is influenced by many factors including, the pH of the reaction medium, the temperature, the hydrogen peroxide concentration and the amount of catalyst which is needed to optimize this process for assessing their measurable performances^34^. Conventional optimization process was determined through the interaction of the individual factors at-a-time while keeping all other conditions constant, this approach does not take into account cross-effects from the factors considered and leads to a poor optimization result^35^. When a multifactor system is present, it is more appropriate to employ statistical-based optimization strategies to achieve such a goal^36^. An experimental design methodology is a modern approach, which has been widely used in several applications^1,37,38^ also allowing the modeling of the process. In fact, the design of experiments (DoE) is used to identify or screen the important factors affecting a process or product and to develop statistically significant empirical models^35,39,40^. To our best knowledge, no study has reported the statistical modeling and optimization of heterogeneous Fenton-like removal of organic pollutant using fibrous catalysts through a full factorial design.

Therefore, in this study, an L^27^ full factorial experimental design was used to optimize the removal process of organic pollutants (dye) by a heterogeneous Fenton-like process. Individual and interaction effects of pH and concentration of hydrogen peroxide [H_2_O_2_]_μl_ was studied in a form of statistical modeling. The responses considered were the reduction of dye concentration (%) after oxidation. Three different catalysts (based on the preparation process) chosen for this study were from our newly developed fibrous catalysts (PET–NH_2_–Fe|PET–Si–NH_2_–Fe|PET–SH–Fe)^20^. Electrokinetic and X-ray photoelectron spectroscopy (XPS) analysis of resultant materials were also studied. Synthesis and structural analysis of zerovalent iron nanoparticles, formation of Fe^2+^, reactive species and detailed mechanism of dye removal can be found in our previous studies^20,33^.

## Experimental

### Materials

Analytical grade polyamidoamine-PAMAM dendrimer, 3-(aminopropyl)triethoxysilane (APTES), 1-thioglycerol (SH), ammonium hydroxide (NH_4_OH); absolute ethanol (Et-OH); sodium borohydride (NaBH_4_), crystal violet (CV), hydrogen peroxide (H_2_O_2_, 30% w/v) and iron nitrate (Fe(NO)_3_) were purchased from Sigma Aldrich Ltd and used as received without any further purifications. Deionized water from Milli-Q^®^ Direct 8 water purification system was used throughout all experiments. The polyester nonwoven membrane used in this study was fabricated in The Swedish School of Textiles (University of Borås) based on a web of cylindrical polyethylene terephthalate fibres (average diameter 12 μm) formed by carding and consolidated by needle punching. The physical characteristics of the fabricated nonwoven are displayed in Table S1.

### Preparation and chemical grafting of PAMAM, APTES, SH on polyester nonwoven fabrics

Polyester nonwoven fabrics underwent multi-step surface functionalization process includes, (a). Plasma activation of polyester surface, (b) Chemical grafting of crosslinkers (PAMAM dendrimer, APTES and SH) and (c) Loading of zerovalent iron nanoparticles through in-situ reduction and immobilization as explained in our previous articles^20,33^. Typically, air atmospheric plasma treatment based on dielectric barrier discharge (DBD) was used to activate the polyester surface using a CoatingStar plasma treatment set-up provided by Ahlbrandt System (Germany) at electrical power of 750 W at a speed of 2 m/min.

PAMAM and APTES were chemically grafted using ethanol/water (3:1 v/v) as solvent under constantly stirring for 4 h (PAMAM in air and APTES in N_2_ chamber) at 70 °C. Following the same protocol, SH was grafted in N_2_ chamber for 12 h. The resulting PET–NH_2_, PET–Si–NH_2_, and PET–SH were filtered, washed and dried at 60 °C overnight.

### Loading of zero-valent iron nanoparticles on polyester nonwoven fabrics

The immobilization of zerovalent iron nanoparticles (Fe-NPs) on PET–NH_2_, PET–Si–NH_2_ and PET–SH were achieved using Fe(NO)_3_ (0.6 wt.%) as precursors and sodium tetrahydroborate (0.9 mM) as a reducing agent. The resultant catalysts, termed as PET–NH_2_–Fe, PET–Si–NH_2_–Fe, and PET–SH–Fe were mildly washed, vacuum dried and stored in a sealed O_2_-free desiccator before further characterization and use.

### Material characterizations

Electrokinetic measurements were carried out to determine the ζ-potential values of fabrics as a function of the pH values of the electrolyte solution (0.001 M KCl). The measurement was carried out using the streaming potential method, in which a liquid is forced to flow through two parallel plates contain samples and streaming potential is generated (Surpass, Anton Paar AB., Sweden). 0.01 M HCl and 0.01 M NaOH were used to adjust the pH values of the electrolyte solution. The ***ζ*** potential values were calculated using the Helmholtz Smoluchowski equation (Eq. 7).

7 $${\upzeta } = \frac{{ dl{\text{str}}}}{{d{\Delta }p}} \times \frac{ \eta }{{\varepsilon \times \varepsilon_{0} }} \times \frac{ L}{A}$$

where *dl/dp* is the slope of streaming current vs. differential pressure, *η* is electrolyte viscosity, *ε* represents the dielectric coefficient of electrolyte, *ε*_*0*_ represents permittivity, *L* is the length of the streaming channel and *A* is the cross-section of the streaming channel.

X-ray photoelectron spectroscopy (XPS) study was carried out by a spectrophotometer (PHI 5500 ESCA, Physical Electronics INC., USA) equipped with monochromatic aluminium (Al) source (photon energy = 1,486.6 eV). Due to the insufficient conductivity, an electron neutralizer was used to compensate for the charge. Survey scan for the compositional evaluation (Energy range of 0–1,100 eV; Step size of 0.4 eV/step), and the narrow scan for the chemical state analysis with selected range for individual elements and step size of 0.1 eV/step was performed^41–44^. Morphological and structural analysis of such fibrous catalysts were extensively studied in our previous reports^20,33^.

### Heterogeneous Fenton-like removal of crystal violet dyes

The heterogeneous Fenton-like removal of crystal violet dye (50 mg/L) using a fibrous catalyst (25 mg/5 mL, PET–NH_2_–Fe, PET–Si–NH_2_–Fe, and PET–SH–Fe) and H_2_O_2_ (100–500 μl/5 ml) was investigated in different pH (5, 7, 9) at 22 ± 2 °C. UV/visible spectrophotometer was used to monitor the removal process. Equilibrium time to attain the maximum dye removal has been detected individually for each catalyst. The minimum time required for complete removal of dye using a particular catalyst through an individual set of experiment was considered as equilibrium time. The pH range in this study was estimated based on our previous investigation on same catalysts^20^. The equilibrium concentration (Q_e_) of crystal violet solution was measured by referring the absorbance calibration curve of the known standard solution, which allowed calculating the relative dye concentration (%) in the reaction chamber based on initial dye concentration. Based on the relative dye concentration in the chamber at the equilibrium point, the efficiency of the catalysts was estimated through a passive approach.

### Design of experiment (DoE) and statistical modeling

Matrix of the experiment’s factors, their levels and response have been presented in Table 1. A full factorial design of experiment combining two factors and three levels for all three fibrous catalysts (PET–NH_2_–Fe, PET–Si–NH_2_–Fe, and PET–SH–Fe) were chosen. According to the number of control parameters and their levels, a distinct full factorial design has been selected (L^27^) to determine the optimal number of experiments in triplicates (See Table 2). In the end, a passive response from each trial run in terms of the relative concentration of dye at equilibrium was recorded. The availed results from each trial run are evaluated in “Minitab^®^ 17 graphical and statistical analysis tool” based on main effects, interaction, ANOVA, and a response table.

**Table 1:** Matrix of experiment’s factors, their levels and response.

Catalyst	Factors	Level			Response
		A	B	C	
PET–NH_2_–Fe	pH (X1)	5	7	9	Dye Conc., Q_e_ (%)
	[H_2_O_2_]_μl_ (X2)	100	300	500	Dye Conc., Q_e_ (%)
PET–Si–NH_2_–Fe	pH(X1)	5	7	9	Dye Conc., Q_e_ (%)
	[H_2_O_2_]_μl_ (X2)	100	300	500	Dye Conc., Q_e_ (%)
PET–SH–Fe	pH(X1)	5	7	9	Dye Conc., Q_e_ (%)
	[H_2_O_2_]_μl_ (X2)	100	300	500	Dye Conc., Q_e_ (%)

*Q*^*e*^ concentration of pollutant at equilibrium.

**Table 2:** L^27^ full factorial design of experiment based on factors, levels and response data [relative dye concentration at equilibrium (%)].

RunOrder	PtType	Blocks	Factors		Relative dye concentration, Q_e_ (%)		
			pH (X1)	[H_2_O_2_] (X2)	PET–NH_2_–Fe	PET–Si–NH_2_–Fe	PET–SH–Fe
1	1	1	5	100	0.8070	0.7501	0.7022
2	1	1	5	300	0.4354	0.5101	0.4469
3	1	1	5	500	0.0109	0.0243	0.0228
4	1	1	7	100	0.9625	0.9512	0.9654
5	1	1	7	300	0.8418	0.8626	0.7384
6	1	1	7	500	0.4209	0.4543	0.4481
7	1	1	9	100	0.9412	0.9837	0.9801
8	1	1	9	300	0.9501	0.9402	0.9619
9	1	1	9	500	0.9459	0.9932	0.9454
10	1	1	5	100	0.8319	0.7978	0.6421
11	1	1	5	300	0.4158	0.5498	0.4106
12	1	1	5	500	0.0108	0.0183	0.0208
13	1	1	7	100	0.9572	0.9403	0.9132
14	1	1	7	300	0.8198	0.7806	0.7002
15	1	1	7	500	0.4351	0.4199	0.4278
16	1	1	9	100	0.9803	0.9967	0.9903
17	1	1	9	300	0.9308	0.976	0.9625
18	1	1	9	500	0.9512	0.952	0.9398
19	1	1	5	100	0.8197	0.8212	0.7233
20	1	1	5	300	0.4637	0.4889	0.3606
21	1	1	5	500	0.0103	0.0199	0.0199
22	1	1	7	100	0.9498	0.9731	0.9573
23	1	1	7	300	0.8219	0.8195	0.7498
24	1	1	7	500	0.4664	0.4821	0.4302
25	1	1	9	100	0.9309	0.971	0.9863
26	1	1	9	300	0.9578	0.9804	0.9512
27	1	1	9	500	0.9141	0.9436	0.9607

## Results and discussions

The results of this study has been divided into two parts; (1) the first part will discuss the statistical modeling and optimization of the process parameters that affect the mean and variance of the attributes and identify the significant contribution and, (2) the second part will discuss the characterization of fibrous catalyst used in this study.

### Part-1: Statistical modeling and optimization of the differences in the process parameters

#### Analysis of main effects

**Figure 1:**
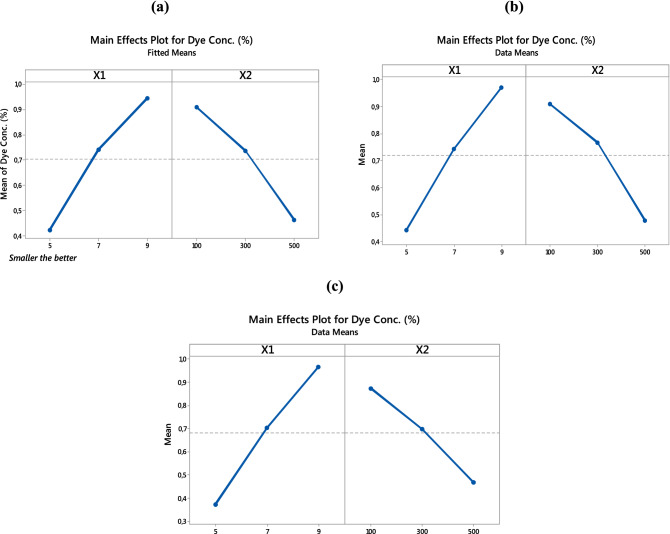
Main effect plots for dye conc. (%) of (**a**) PET–NH_2_–Fe, (**b**) PET–Si–NH_2_–Fe and (**c**) PET–SH–Fe; Smaller the better ; [Response: Dye concentration (%)].

The main effect is defined as the effect of an independent variable on a dependent variable averaged across the levels of all independent variable. By analyzing the main effects of the parameters, the general trends of the factor’s influence on the process can be determined^45^. Figure 1 shows the main effect plots for the relative dye concentration (%) after Fenton-like oxidation using PET–NH_2_–Fe, PET–Si–NH_2_–Fe and PET–SH–Fe based on the date shown in Table 2. Results shown in Fig. 1a–c implies that, regardless of the type of the catalysts, with the changing in level in both X1 and X2, the response has significantly altered in a linear model which validates the statistically significant effect of pH and [H_2_O_2_]_μl_ on the response. Concentration may negatively affect the removal process as explained in various reports^8,20,46,47^.

Detailed analysis of the results shows that the lowest level of the factor X1 and highest levels of the factor X2 determined the maximum removal efficiency. With the increase in pH, the net negative surface charges on the catalysts surface cancelling the efficiency of reactive species that are responsible for the removal. Jung et al. (2013) reported that alkaline medium affects both reagents in the Fenton process causing instability in hydrogen peroxide and forming oxides on iron which significantly hinder the general of reactive species^22^. Lower concertation of hydrogen peroxide is too concentrated to react with mineralized iron to produce reactive species. However, it also needs to be noted that, after the saturation concentration of reactants, a further increase in concentration may negatively affect the removal process as explained in various reports^8,20,46,47^.

#### Analysis of interaction effects between factors

**Figure 2:**
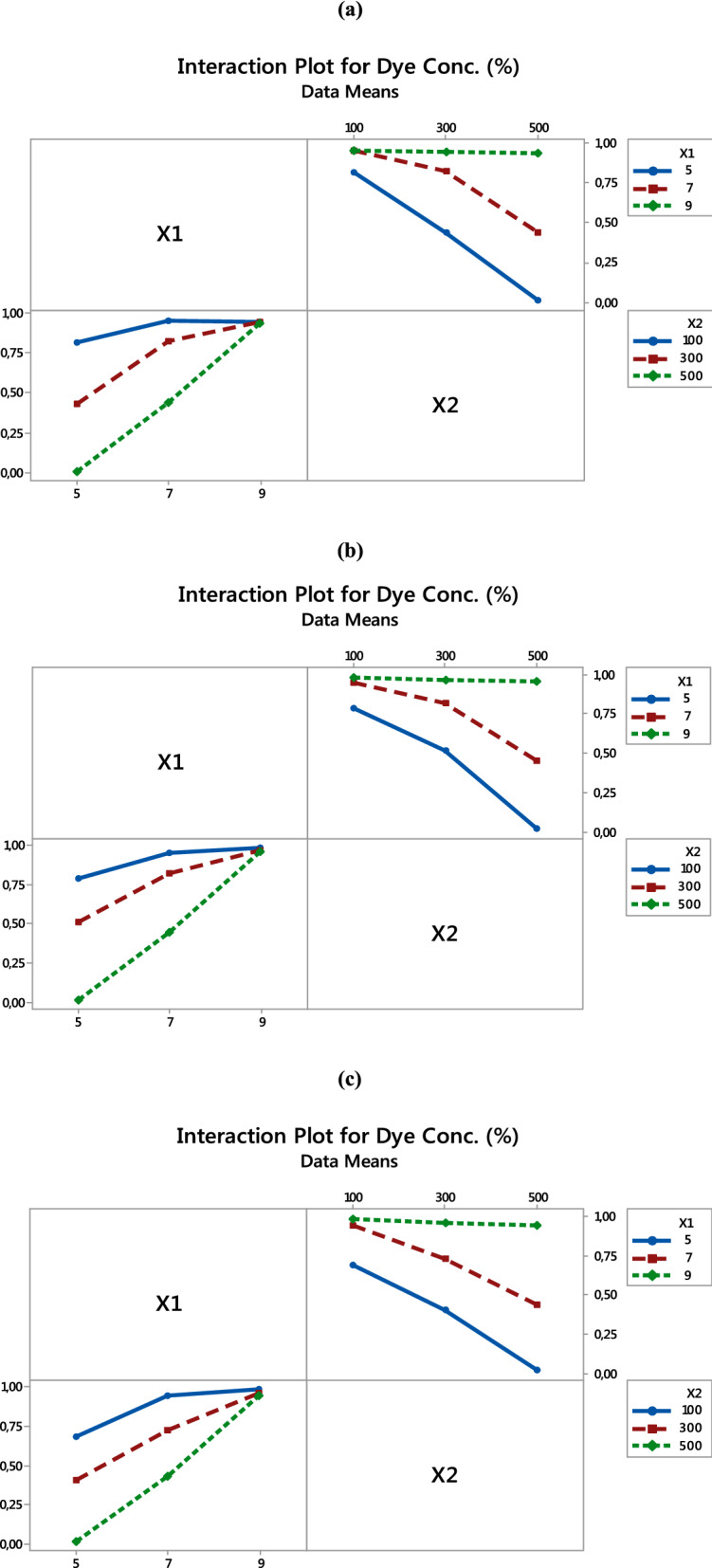
Interaction plots for dye conc. (%) of (**a**) PET–NH_2_–Fe, (**b**) PET–Si–NH_2_–Fe and (**c**) PET–SH–Fe [Response : Dye concentration (%)].

Figure 2 shows the interaction effects of the factors in the low level and the high level of another factor. Figure 2a shows the interaction plots for PET–NH_2_–Fe for the factors pH and [H_2_O_2_]_μl_. It can be seen that the interactions between X1_A×B_ and X2 _B×C_ have significance among factors (see Fig. 2a). The remaining interaction X1_C_ and X2_A_ show near straight effect, forming a parallel-like line, meaning these two conditions do not have a relation. On the contrary, the interaction between X1_A_ and X2_C_ has shown the most significant relationship. According to Hu, J. et al. (2019), an interaction exists if the relationship is represented by nonparallel lines while parallel lines denote no relationship between the parameters^47^. The interaction plots of other samples (PET–Si–NH_2_–Fe and PET–SH–Fe) show similar trends like PET–NH_2_–Fe (See Fig. 2b,c). The overall scenario concludes that the removal efficiency increases with the increase in the concentration of hydrogen peroxide [H_2_O_2_] at the same time decrease in pH also increases removal efficiency as consistent with main effects. This also indicates that the interaction plots are suitable for exploring the process parameters.

#### Analysis of variance-ANOVA

The main effect and interaction plots provide the optimal levels of each factor but do not indicate which factor has the most significant impact on the output and the contribution of each factor. This was determined by the ANOVA, which is a robust method to determine the contribution of each factor and the significance of the optimization model^48^. It is accomplished by determining the Fischer’s test value (F-value) and the sum of squares, which are used to evaluate the significance of the parameters; p values below 0.05 or 5% were considered statistically significant^49^. Tables 3, 4 and 5 shows the ANOVA results for the PET–NH_2_–Fe, PET–Si–NH_2_–Fe and PET–SH–Fe, respectively. Results indicate, among the significant parameters, the pH has a large influence on the efficiency of the removal with an F-value of 2045.59, 866.65 and 1314.62 and a p-value of 0.000 (same for all three) for ET–NH_2_–Fe, PET–Si–NH_2_–Fe and PET–SH–Fe, respectively. The concentration of hydrogen peroxide [H_2_O_2_] is also significant (p = 0.000) but has a lower influence than that of pH with F-values of 1496.75, 594.62 and 615.72, respectively. The model summary shows strong correlation as expressed by R^2^ value over 98% for all three catalysts (see Tables S2, S4, S6). On the other hand, by comparing the F-values among three fibrous catalysts considering all factors and levels, it can be seen that the type of catalyst does not have any significant impact on the dye removal. The 2-way interaction of the factors also shows an overall significant impact on dye removal. However, extensive analysis (see Tables S3, S5, S7) showing coefficient of each interaction identifies the interaction between X1_B_ and X2_A_ as not significant in PET–Si–NH_2_–Fe (see Table S5), X1_A_ and X2_B_ plus X1_B_ and X2_B_ in PET–SH–Fe (see Table S7).

**Table 3:** Analysis of variance-ANOVA for dye conc. (%) [PET–NH_2_–Fe].

Source	DF	Seq SS	p%	Adj SS	Adj MS	F-value	p-value	Remarks
Model	8	2.66076	99.79	2.66076	0.332595	1,092.32	0.000	Significant
Linear	4	2.15717	80.91	2.15717	0.539293	1771.17	0.000	Significant
X1	2	1.24570	46.72	1.24570	0.622849	2045.59	0.000	Significant
X2	2	0.91147	34.19	0.91147	0.455737	1,496.75	0.000	Significant
2-Way interactions	4	0.50359	18.89	0.50359	0.125897	413.48	0.000	Significant
X1*X2	4	0.50359	18.89	0.50359	0.125897	413.48	0.000	Significant
Error	18	0.00548	0.21	0.00548	0.000304

**Table 4:** Analysis of variance-ANOVA for dye conc. (%) [PET–SI–NH_2_–Fe].

Source	DF	Seq SS	p%	Adj SS	Adj MS	F-value	p-value	Remarks
Model	8	2.58356	99.49	2.58356	0.322945	442.61	0.000	Significant
Linear	4	2.13239	82.12	2.13239	0.533098	730.63	0.000	Significant
X1	2	1.26468	48.70	1.26468	0.632338	866.65	0.000	Significant
X2	2	0.86772	33.42	0.86772	0.433858	594.62	0.000	Significant
2-Way interactions	4	0.45117	17.37	0.45117	0.112793	154.59	0.000	Significant
X1*X2	4	0.45117	17.37	0.45117	0.112793	154.59	0.000	Significant
Error	18	0.01313	0.51	0.01313	0.000730

**Table 5:** Analysis of variance-ANOVA for dye conc. (%) [PET–SH–Fe].

Source	DF	Seq SS	p%	Adj SS	Adj MS	F-value	p-Value	Remarks
Model	8	2.65499	99.59	2.65499	0.331873	550.48	0.000	Significant
Linear	4	2.32752	82.31	2.32752	0.581880	965.17	0.000	Significant
X1	2	1.58511	59.46	1.58511	0.792555	1,314.62	0.000	Significant
X2	2	0.74241	27.85	0.74241	0.371205	615.72	0.000	Significant
2-Way interactions	4	0.32747	12.28	0.32747	0.081867	135.79	0.000	Significant
X1*X2	4	0.32747	12.28	0.32747	0.081867	135.79	0.000	Significant
Error	18	0.01085	0.41	0.01085	0.000603			

The influence of each factor is also expressed as the percent contribution (p%). From the results, it can be seen that pH has the percent contribution of 46.72% in PET–NH_2_–Fe, 48.70% in PET–Si–NH_2_–Fe and 51.46% in PET–SH–Fe, followed by the [H_2_O_2_]_μl_ where 34.19% in PET–NH_2_–Fe, 33.42% in PET–Si–NH_2_–Fe and 27.456% in PET–SH–Fe was recorded. These findings indicate that, although both factors have significant influence over the dye removal efficiency, pH has a higher influence than that of [H_2_O_2_]_μl_. Seq. SS, Adj. SS, Adj. MS values are in line with f-values of all fibrous catalysts and all factors. A detailed study among three catalysts indicates that the influences of the pH are greatly affecting on by PET–SH–Fe followed by PET–Si–NH_2_ and PET–NH_2_–Fe whereas the influence of [H_2_O_2_]_μl_ largely in PET–NH_2_–Fe followed by PET–Si–NH_2_–Fe and PET–SH–Fe catalyst. The prominent effect of pH over [H_2_O_2_]_μl_ can be explained by the synergic effect of pH on stabilization of hydrogen peroxide and formation of reactive species, which ultimately performs the removal of dyes, in an unlikely pH condition the no removal occurs, whereas in a favourable pH condition, lower or higher concentration of [H_2_O_2_]_μl_ will affect the removal but will not be absent^46,50^.

#### Analysis of residual plots

**Figure 3:**
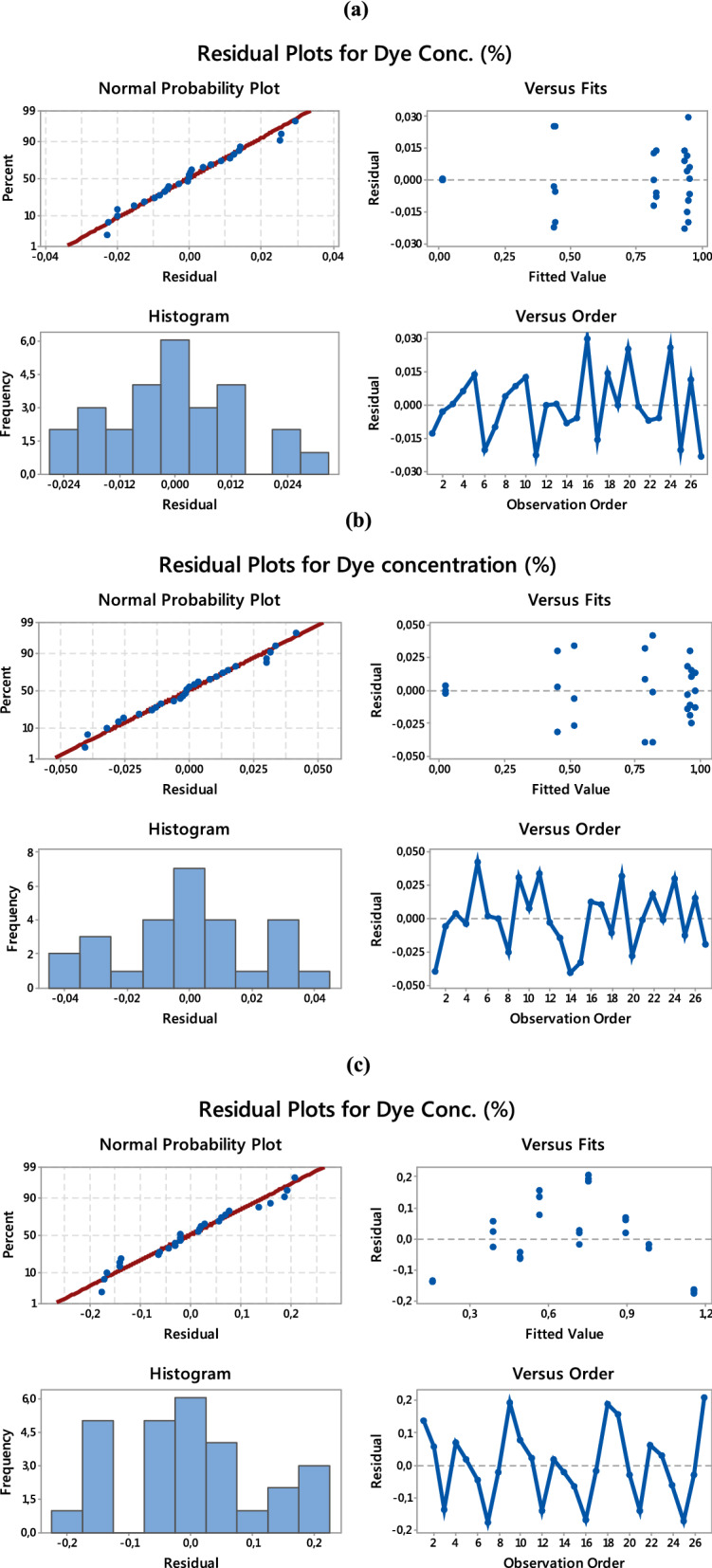
Residual plots for dye conc. (%) (**a**) PET–NH_2_–Fe, (**b**) PET–Si–NH_2_–Fe and (**c**) PET–SH–Fe.

Figure 3a–c shows the residual plots for dye concentration (%) for PET–NH_2_–Fe, PET–Si–NH_2_–Fe and PET–SH–Fe fibrous catalysts, respectively. The four plots of normal probability, residuals versus the fitted values, the histogram, and the residuals versus the observation order are shown in Fig. 3. From Fig. 3a, it is evident that the normal probability plot exhibits a nearly linear response, demonstrating that the errors are distributed normally. This is confirmed by the histogram, which also shows a normal distribution. The plot of the residuals versus the fitted values indicates that the residuals are randomly distributed around the zero lines, indicating that no sequential association exists and the errors have a constant variance. Moreover, the plot of the residuals versus the observation order is used to evaluate the pattern that may have an impact on the output. It is observed that the residuals are normally distributed close to the zero lines, which implies that there is a fair association with the parameters that do not require any further analysis of the errors^51–53^. A similar trend has been found in Fig. 3b,c.

#### Analysis of individual value plots

**Figure 4:**
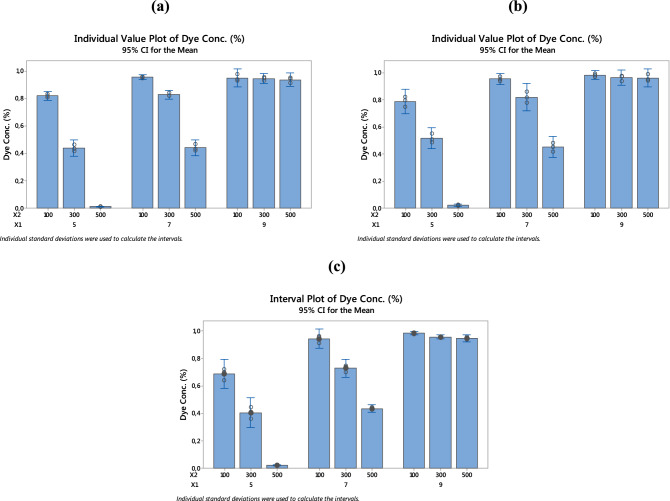
Individual value plots for dye conc. (%); (**a**) PET–NH_2_–Fe, (b) PET–Si–NH_2_–Fe and (**c**) PET–SH–Fe.

Because individual value plots display all values for all groups at the same time, they are especially helpful when the study is dealing with variables, groups, and even subgroups. The individual value plot of dye concentration (%) after oxidation with PET–NH_2_–Fe, PET–SI–NH_2_–Fe and PET–SH–Fe fibrous catalysts, has been plotted for each factor and their levels (see Fig. 4). A close look at all the figures shows that there is a similar trend in all samples indicating the sample type does not influence the response. However, from Fig. 1a it can clearly be seen that both pH and [H_2_O_2_]_μl_ affect the results significantly. With lower pH, all concentration of [H_2_O_2_]_μl_ gives a nominal removal performance, whereas the highest concentration of [H_2_O_2_]_μl_ gives the best efficiency. On the other hand, in pH-7 the efficiency disrupted significantly by more than 50%. And finally, in the highest level of pH, there is no removal recorded regardless of the change in concentration of hydrogen peroxide. The same explanation corresponds to the other catalysts samples. These results provide significant statistical evidence to predict the best possible parameters and their levels.

#### Analysis of fitted line plots

**Figure 5:**
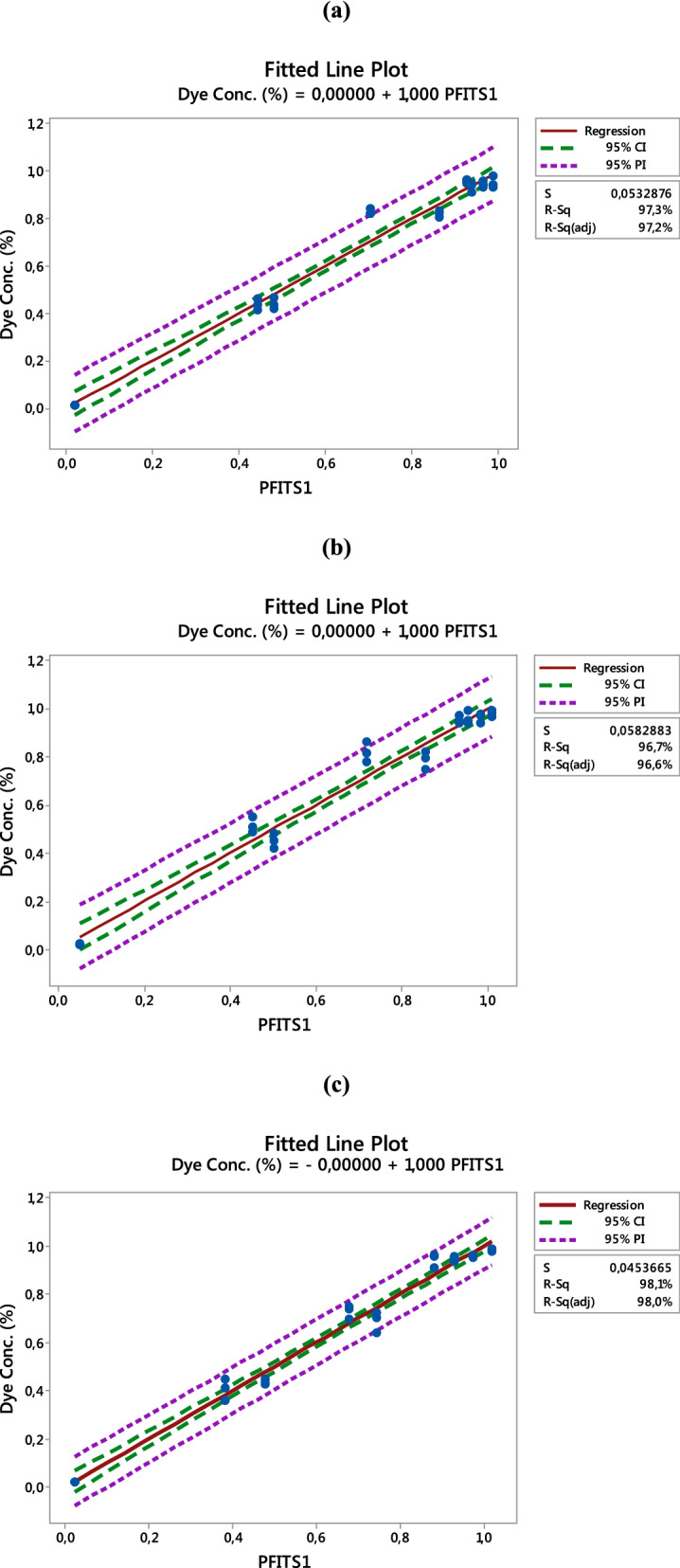
Fitted plots for dye Conc. (%); (**a**) PET–NH_2_–Fe, (**b**) PET–Si–NH_2_–Fe and (**c**) PET–SH–Fe.

Figure 5a–c shows the fitted plots of the predicted versus the actual values for the responses for PET–NH_2_–Fe, PET–Si–NH_2_–Fe and PET–SH–Fe fibrous catalysts, respectively. It can be observed that the points reflect the deviation of the actual values from the predicted or fitted values. The graph indicates that the model is significant because the residuals are close to the diagonal line. In addition, the Pearson correlation coefficient between the predicted and actual values for the responses was 1.000 with a p-value of 0.000, which indicates a strong relationship between the predicted and actual values for all three fibrous catalysts^54,55^.

### Part-2: Characterization of fibrous catalysts

#### Electrokinetic measurement (ζ-potential analysis)

**Figure 6:**
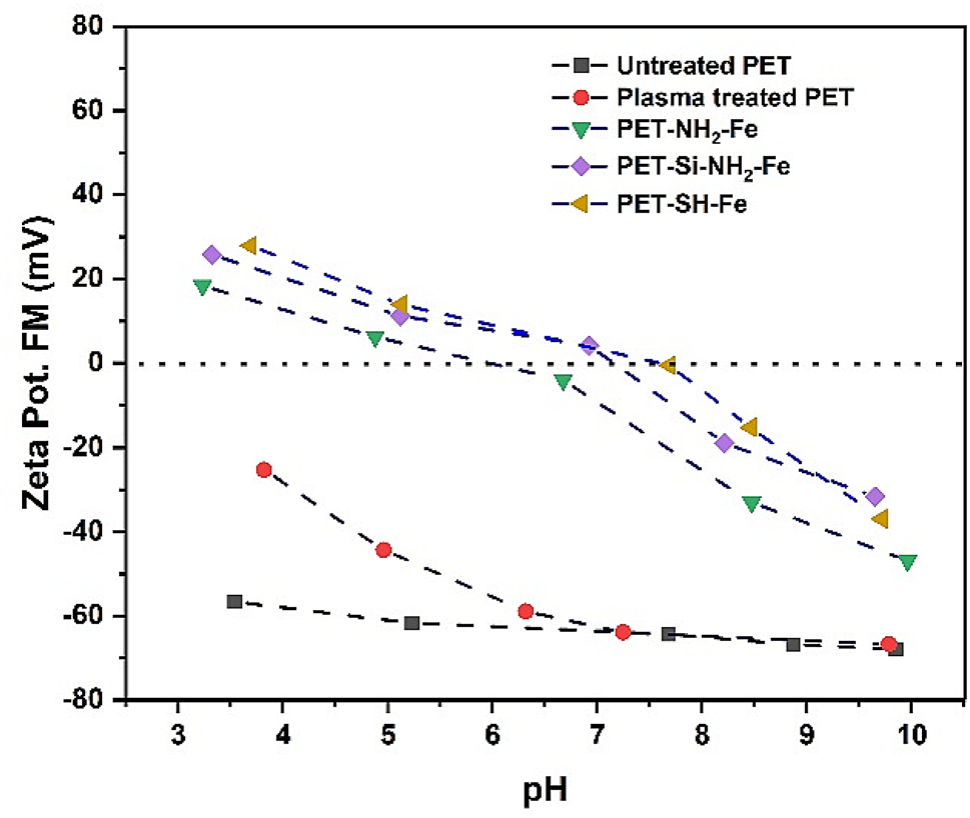
The ζ-potential value of samples as a function of the pH of the electrolyte solution (0.001 M KCl).

The isoelectric point (iep = pH|ζ = 0) can be determined through the ***ζ***-potential values of untreated, plasma-treated and iron-loaded polyester fabrics that has been observed by electrokinetics and measured as streaming potential. The summary of the ***ζ***-potential analysis of the samples is displayed in Fig. 6. It can be seen that the isoelectric point of untreated polyester fibres is pH = 3.9 which means that negative surface charge will be observed for the polyester fibres for pH higher than 3.9. Due to the further addition of –OH and –COOH groups during plasma treatment of the PET fibres (PET), the surface charge and isoelectric point were further negative starting from − 6.89 mV at pH = 3.45 to until − 49.23 mV at pH = 9.91 (see Fig. 6). With a negative surface containing –OH and –COOH groups may pose an ideal environment for robust electrostatic incorporation of cationic iron ions (Fe^3+^) followed by reduction and immobilization into polyester fabrics. Upon incorporation of Fe^0^, the isoelectric point of PET–NH_2_–Fe, PET–Si–NH_2_–Fe and PET–SH–Fe shifted to pH around 7 signifies (see Fig. 6) an increase in ***ζ***-potential which can be due to the loading of iron particles and reduction of oxygen-containing groups.

#### X-ray photoelectron spectroscopy (XPS)

**Figure 7:**
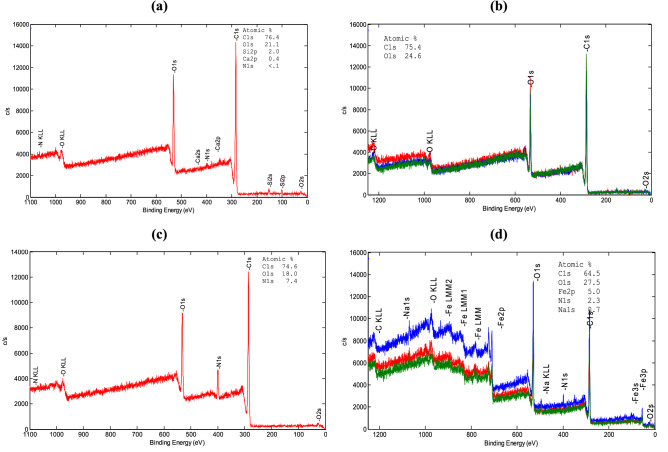
Wide scan XPS spectra of (**a**) untreated PET, (**b**) Plasma treated PET, (**c**) PET–NH_2_ and (**d**) PET–NH_2_–Fe.

The element composition and the nature of the chemical bonding of a plasma treated PET and PET-NH_2_-Fe were evaluated by XPS (see Fig. 7 and Fig. S2 of supplementary information). Results summarized in Table 6 shows that a control sample contains 76.4 a.t% carbon and 21.1 a.t% oxygen, which after plasma activation changed into 75.4 a.t% carbon and 24.6 a.t% oxygen indicating an increase in oxygen due to formation of –COOH and –OH groups on the fibre surface. After the modification of the fabric with PAM (PET–NH_2_), nitrogen was detected in the surface composition having and iron was detected on PET–NH_2_–Fe sample.

**Table 6:** Atomic proportion (a.t%) of the surface chemical composition of the samples.

Sample	C (a.t %)	O (a.t %)	N (a.t %)	Fe (a.t %)
Untreated PET	76.4	21.1	< 0.1	0
Plasma treated PET	75.4	24.6	0	0
PET–NH_2_	74.6	18.0	7.4	0
PET–NH_2_–Fe	64.5	27.5	2.3	5.0

A detailed study of Fig. 7b (plasma-treated sample) illustrates that the sample consists mainly of C and O in a ratio around 3:1. Majority of the carbon (i.e. 60% of the area) refers to the C≡C and C=C bonding type in the hydrocarbon chain, whereas 40% of the area corresponds to C=O, C–O and COO^−^. O1s, on the other hand, shows 90% of the signal area is for C=O, C–O and –OH. All features in the O1s and part of C1s correspond to polyester, i.e. the COO^-^ functional group. Figure 7c and Fig. S2d shows the presence of 7.4 at.% nitrogen that confirms the grafting of PAM is mainly a graphitic-N type (R_3_N) and the rest refers to pyrrolic (R_2_NH). C–O proportion is enriched in C1s and O1s peaks. Figure 7d and Fig. S2f indicates that the sample consists of 5.0 at.% Fe in Fe(III) state. 2.3 at.% N at 400.0 eV corresponds to a 100% pyrrolic-N, secondary amine and/or primary amide condition, i.e. C_4_H_9_N/R_2_NH/RCONH_2_). The C–O ratio is around 2:1. Alkane-type hydrocarbon chain, i.e. C1s = 284.8 eV (C–C) is determined. Rest of the C signal can be assigned to the carboxylic group, i.e. COO^−^. Moreover, most of the deconvoluted peaks in O1s also agrees with the features in a carboxylic group. In addition, a deconvoluted located at 529.5 eV corresponds to metal, could be metal-Fe (zerovalent iron).

## Conclusions

A full factorial design of experiment to predict and optimize the heterogeneous Fenton-like removal of organic pollutants using newly developed fibrous catalysts has been successfully achieved. The statistical insight on the effect of the factors (X1 and X2) and their levels in the removal process has been identified. Primarily, the statistical model showed that for both factors (X1 and X2) optimum values for the process variables exist. The results have been summarized below.

- Optimum operating conditions were determined based on a mathematical model in the form of statistical modeling, they are: initial acidic pH of 5, [H_2_O_2_]_μl_ of 500 μl which is a distinctive behaviour in the heterogeneous Fenton-like process. Under these conditions, 99% of pollutants are being removed in a rapid oxidation reaction.
- The interaction between X1 and X2 was of statistical significance with respect to the lower level of X1 and a higher level of X2 whereas the X1 turned into the key factor for the process. Whereas the type of catalysts turned out to be statistically insignificant.
- The ANOVA results provided evidence of the statistical significance of the factors and their corresponding levels that gives clear information during optimizing the process productivity and effective prediction.
- The ζ-potential and XPS analyses of the fibrous catalysts validated the surface modification of polyester fabric and loading of zerovalent iron nanoparticles.

While providing valuable insight into the roles of factors, the model provides rational ground to undertake a kinetic model that will further provide an in-depth understanding of the mechanisms and kinetics of the heterogeneous Fenton-like removal of complex pollutants in wastewater using such catalysts.

## Supplementary information

Supplementary file1
