# Supplementary material for: Statistical modeling and optimization of heterogeneous Fenton-like removal of organic pollutant using fibrous catalysts: a full factorial design
Source: Sci Rep. 2020 Sep 30;10:16133. doi: 10.1038/s41598-020-72401-z (PMC7528022; doi:10.1038/s41598-020-72401-z)
Supplement: Supplementary file 1 — Supplementary file1 [file 41598_2020_72401_MOESM1_ESM.pdf]

-------------Supplementary information--------------

Statistical modeling and optimization of heterogeneous Fenton-like removal of organic pollutant using fibrous catalysts: A full factorial design

Mohammad Neaz Morshed [1, 2, 3, 4*], Md. Nahid Pervez[5], Nemeshwaree Behary[2, 3], Nabil Bouazizi[2], Jinping Guan[4] and Vincent A. Nierstrasz[1]

[1] *Textile Material Technology, Department of Textile Technology, Faculty of Textiles, Engineering and Business, University of Borås, SE-50190, Borås, Sweden*

[2] *Ecole Nationale Supérieure des Arts et Industries Textiles (ENSAIT), GEMTEX Laboratory, 2 allée Louise et Victor Champier BP 30329, 59056 Roubaix, France*

[3] *Université de Lille, Nord de France, F-59000 Lille, France*

[4] *College of Textile and Clothing Engineering, Soochow University, 215006 Suzhou, China*

[5] *Swedish Centre for Resource Recovery, Faculty of Textiles, Engineering and Business, University of Borås, SE-50190, Borås, Sweden*

* *Corresponding author´s email address: mohammad_neaz.morshed@hb.se*

1. EXPERIMENTAL

Table S1: Physical characteristics of polyester nonwoven membranes

**Physical characteristics**	**Values**
Mass per unit area (g/m^2)	98.00
Thicknesses (mm)	0.94
Fibre density	0.80
Porosity (%)	99.91
Air permeability (mm/s)	854.20

Prior to surface activation, the dirt, dust, contaminants and spinning oil of the fabricated polyester nonwoven membranes were removed by a series of cleaning steps including Soxhlet extraction method as described V. Takke et. al (2009) and others [1-3]. The removal of impurities was done in three (03) folds and successively assessed by analyzing surface tension of rinsed water (from PET rinsing bath) with freshwater (surface of freshwater is 72.6 mN/m, The closest surface tension of the rinsed water to freshwater refers to the high degree of cleanliness) [4].

2. RESULTS

2.1. Analysis of variance (ANOVA)

Table S2: Model Summary [PET-PAM-Fe]

**Standard deviation, S**	**R-sq**	**R-sq(adj)**	**PRESS**	**R-sq(pred)**
0.0174495	99.79%	99.70%	0.0123316	99.54%

Table S3: Coefficients [PET-PAM-Fe]

Term	Coef	SE Coef	95% CI	T-Value	P-Value	Remarks
**Constant**	0.70308	0.00336	(0.69603; 0.71014)	209.37	0.000	Significant
**X1**						
5	-0.28025	0.00475	(-0.29023; -0.27027)	-59.01	0.000	Significant
7	0.03863	0.00475	(0.02865; 0.04861)	8.13	0.000	Significant
9	0.24162	0.00475	(0.23164; 0.25160)	50.88	0.000	Significant
**X2**						
100	0.20586	0.00475	(0.19589; 0.21584)	43.35	0.000	Significant
300	0.03437	0.00475	(0.02440; 0.04435)	7.24	0.000	Significant
500	-0.24024	0.00475	(-0.25021; -0.23026)	-50.59	0.000	Significant
**X1*X2**						
5*100	0.19084	0.00672	(0.17673; 0.20495)	28.41	0.000	Significant
5*300	-0.01891	0.00672	(-0.03302; -0.00480)	-2.82	0.011	Significant
5*500	-0.17193	0.00672	(-0.18604; -0.15782)	-25.60	0.000	Significant
7*100	0.00893	0.00672	(-0.00518; 0.02304)	1.33	0.000	Significant
7*300	0.05175	0.00672	(0.03764; 0.06586)	7.70	0.000	Significant
7*500	-0.06067	0.00672	(-0.07478; -0.04656)	-9.03	0.000	Significant
9*100	-0.19976	0.00672	(-0.21387; -0.18565)	-29.74	0.000	Significant
9*300	-0.03284	0.00672	(-0.04695; -0.01873)	-4.89	0.000	Significant
9*500	0.23260	0.00672	(0.21849; 0.24671)	34.63	0.000	Significant

Table S4: Model Summary [PET-APTES-Fe]

Standard deviation. S	R-sq	R-sq(adj)	PRESS	R-sq(pred)
0.0270118	99.49%	99.27%	0.0295503	98.86%

Table S5: Coefficients [PET-APTES-Fe]

Term	Coef	SE Coef	95% CI	T-Value	P-Value	Remarks
**Constant**	0.71855	0.00520	(0.70763; 0.72947)	138.22	0.000	Significant
**X1**						
5	-0.27628	0.00735	(-0.29173; -0.26084)	-37.58	0.000	Significant
7	0.02407	0.00735	(0.00863; 0.03952)	3.27	0.000	Significant
9	0.25221	0.00735	(0.23676; 0.26765)	34.31	0.000	Significant
**X2**						
100	0.19091	0.00735	(0.17546; 0.20635)	25.97	0.000	Significant
300	0.04902	0.00735	(0.03357; 0.06446)	6.67	0.000	Significant
500	-0.23993	0.00735	(-0.25537; -0.22448)	-32.64	0.000	Significant
**X1*X2**						
5*100	0.1565	0.0104	(0.1347; 0.1784)	15.06	0.000	Significant

5*300	0.0250	0.0104	(0.0031; 0.0468)	2.40	0.027	Significant
5*500	-0.1815	0.0104	(-0.2034; -0.1597)	-17.46	0.000	Significant
7*100	0.0213	0.0104	(-0.0005; 0.0432)	2.05	0.055	Not Significant
7*300	0.0293	0.0104	(0.0074; 0.0511)	2.81	0.011	Significant
7*500	-0.0506	0.0104	(-0.0724; -0.0288)	-4.87	0.000	Significant
9*100	-0.1779	0.0104	(-0.1997; -0.1560)	-17.11	0.000	Significant
9*300	-0.0542	0.0104	(-0.0761; -0.0324)	-5.22	0.000	Significant
9*500	0.2321	0.0104	(0.2103; 0.2539)	22.32	0.000	Significant

Table S6: Model Summary [PET-SH-Fe]

**Standard deviation, S**	**R-sq**	**R-sq(adj)**	**PRESS**	**R-sq(pred)**
0.0245536	99.59%	99.41%	0.0244166	99.08%

Table S7: Coefficients [PET-SH-Fe]

**Term**	**Coef**	**SE Coef**	**95% CI**	**T-Value**	**P-Value**	**Remarks**
**Constant**	0.67992	0.00520	(0.66999; 0.68985)	143.89	0.000	Significant
**X1**						
5	-0.30779	0.00668	(-0.32182; -0.29375)	-46.06	0.000	Significant
7	0.02346	0.00668	(0.00942; 0.03750)	3.51	0.002	Significant
9	0.28433	0.00668	(0.27029; 0.29837)	42.55	0.000	Significant
**X2**						
100	0.19344	0.00668	(0.17940; 0.20748)	28.95	0.000	Significant
300	0.01809	0.00668	(0.00405; 0.03213)	2.71	0.014	Significant
500	-0.21153	0.00668	(-0.22557; -0.19749)	-31.65	0.000	Significant
**X1*X2**						
5*100	0.12363	0.00945	(0.10377; 0.14348)	13.08	0.000	Significant
5*300	0.01581	0.00945	(-0.00405; 0.03566)	1.67	0.112	Not Significant
5*500	-0.13944	0.00945	(-0.15929; -0.11958)	-14.75	0.000	Significant
7*100	0.04849	0.00945	(0.02863; 0.06834)	5.13	0.000	Significant
7*300	0.00800	0.00945	(-0.01186; 0.02785)	0.85	0.409	Not Significant
7*500	-0.05648	0.00945	(-0.07634; -0.03663)	-5.98	0.000	Significant
9*100	-0.17211	0.00945	(-0.19197; -0.15226)	-18.21	0.000	Significant
9*300	-0.02380	0.00945	(-0.04366; -0.00395)	-2.52	0.021	Significant
9*500	0.19592	0.00945	(0.17606; 0.21577)	20.73	0.000	Significant

2.2. *Analysis of pareto chart of standardized effects*

(a)

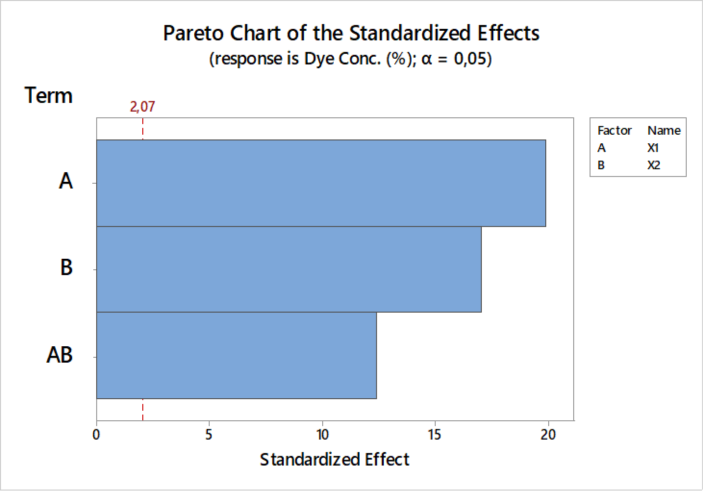

(b)

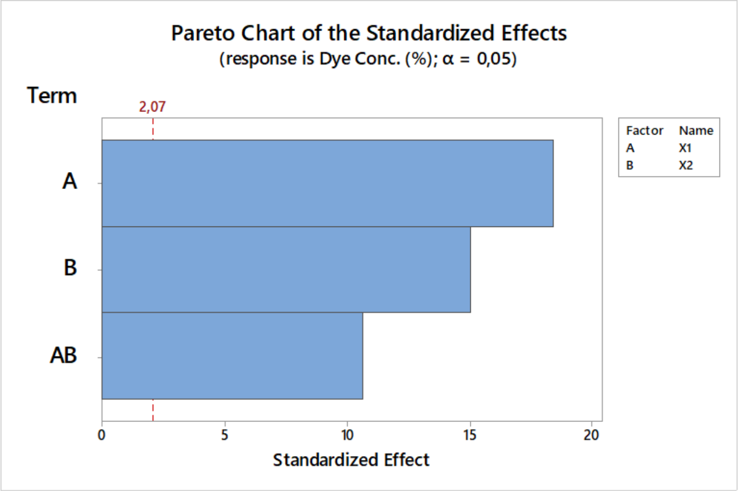

(c)

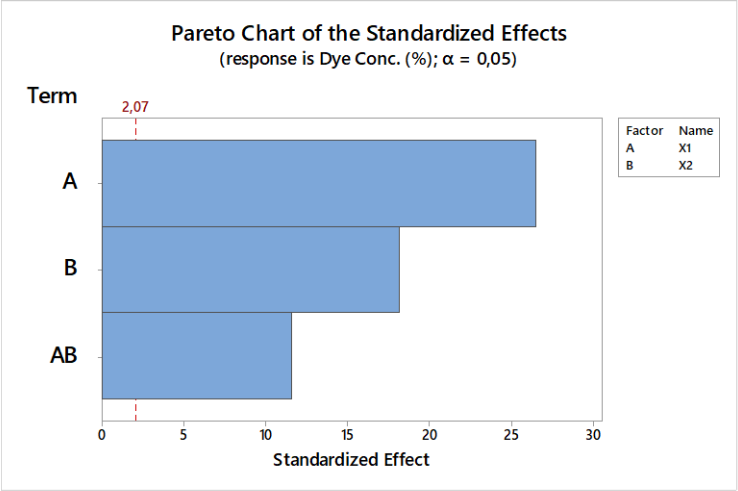

Fig. S1: Pareto chart plots for Dye Conc. (%); (a) PET-PAM-Fe, (b) PET-APTES-Fe and (c) PET-SH-Fe.

2.3. X-ray photoelectron spectroscopy (XPS) analysis

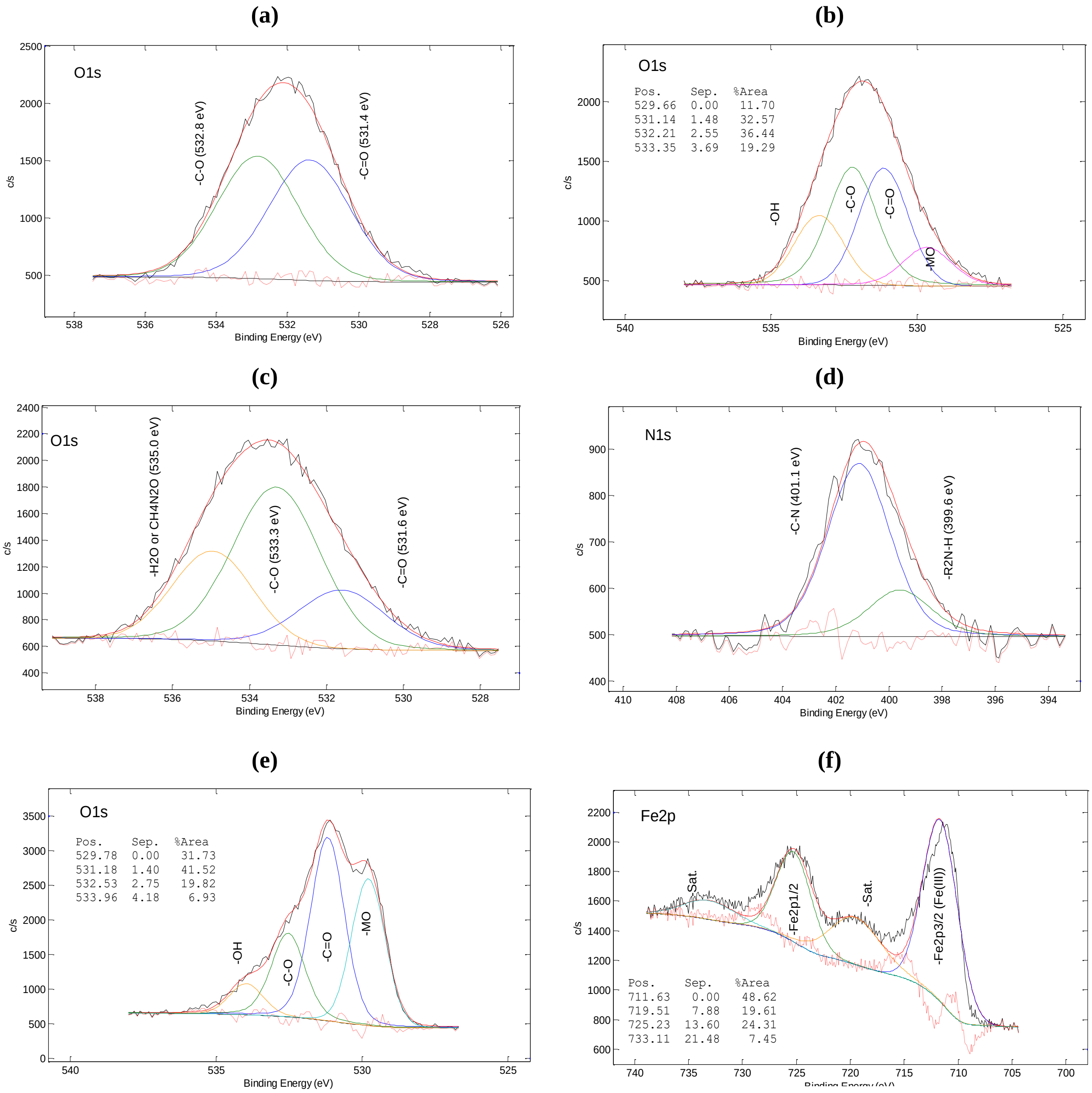

Fig. S2: (a) O1s spectra of untreated PET, (b) O1s spectra of plasma treated PET, (c) O1s spectra of PET-PAM, (d) N1s spectra of PET-PAM, (e) O1s spectra of PET-PAM-Fe and (f) Fe2P spectra of PET-PAM-Fe.

2.4. Structiral analysis of ZVI

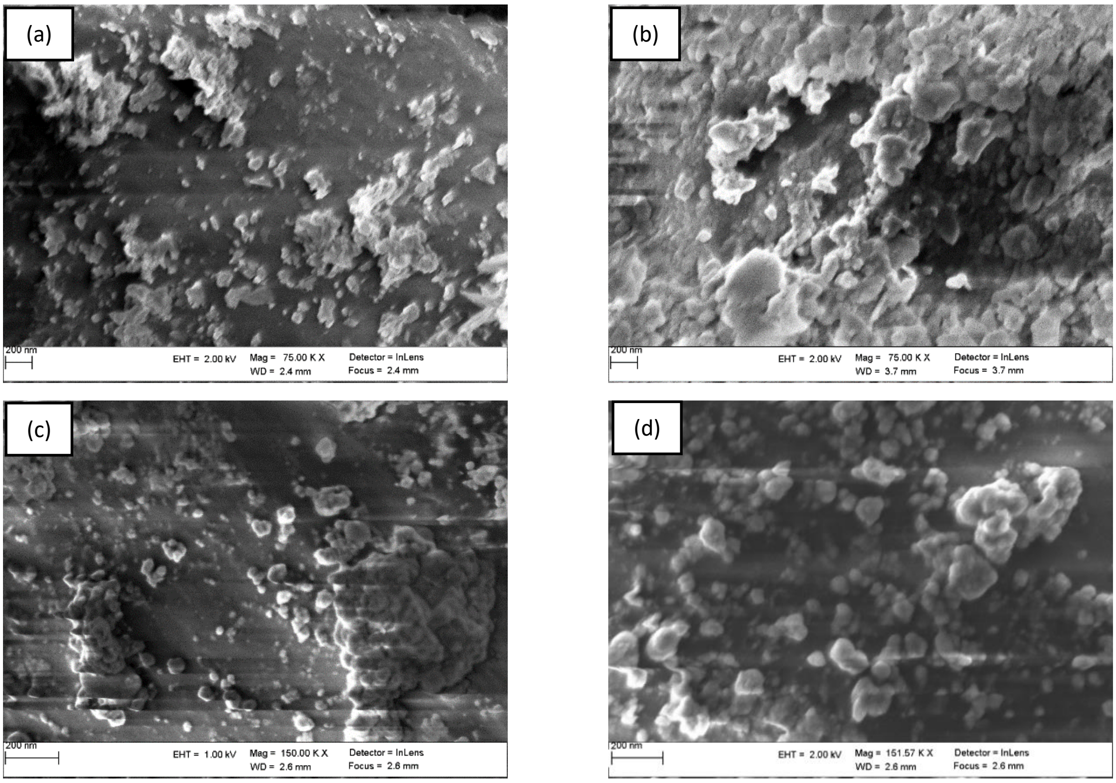

Fig. S3: HR-SEM images of (a) PET-Fe; (b) PET-NH_2-Fe; (c) PET-Si-NH_2-Fe and (d) PET-SH-Fe. *[Same as: 10.1039/d0ra01362e]*

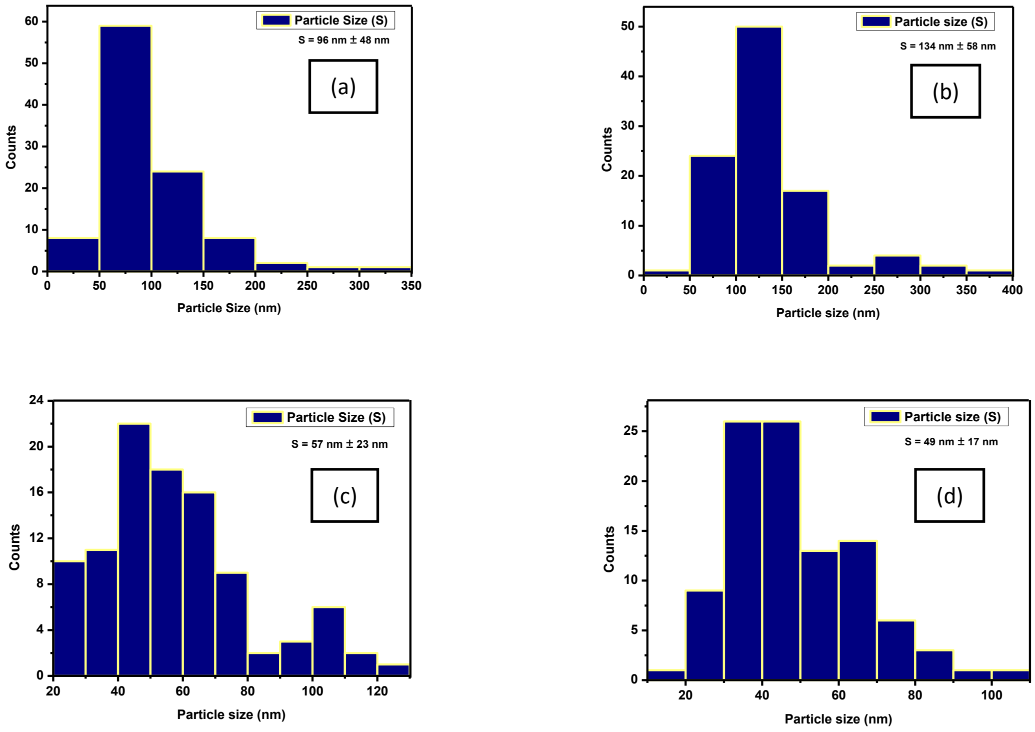

Fig. S4: Particle size distribution histogram of immobilized iron on (a) PET-Fe; (b) PET-NH_2-Fe; (c) PET-Si-NH_2-Fe and (d) PET-SH-Fe. *[Same as: 10.1039/d0ra01362e]*

Surface morphologies of polyester membranes after immobilization of Fe-NPs has been investigated through scanning electron microscopy (SEM). Changes in fibre diameter, particles size of iron has been studied from SEM images through image processing software (ImageJ). SEM images in Fig. S3 shows the presence of iron particle dispersed over the surface of all polyester membranes (PET-Fe; PET-NH2-Fe; PET-Si-NH2-Fe; PET-SH-Fe). This visual evidence provides the proof of successful immobilization of iron nanoparticles on the fibre surface. However, a close look at images reveals that individual pre-treatment of polyester fabric before iron immobilization resulted in variation in dispersion, size and stability of the iron particles. The size distribution histogram of the iron particles (see Fig. S4 and Table S8) shows an irregular distribution of iron particles with an average size ranges from 50-150 nm.

Table S8: Summary of particle size and wt. % of Fe-NPs immobilized on the polyester membrane

Sample	The particle size of Fe-NPs (nm)	mg % of Fe-NPs immobilized
PET-Fe	96 ± 48	18.94
PET-NH_2-Fe	134 ± 58	19.02
PET-Si-NH_2-Fe	57±23	24.12
PET-SH-Fe	49 ± 17	26.10

2.5. *The postulated mechanism of formation of Fe^{2+},the generation of reactive oxidant species and dye removal*

Characteristics property of zerovalent iron (immobilized in the polyester membrane) to produce iron ions followed by participation in Fenton-like reaction in presence of hydrogen peroxide generates high oxidation potential of hydroxyl free radical (The hydroxyl radical (•OH) has a standard oxidation-reduction potential of 2.8 V) (see **Fig. 5** and ***Reaction 1-4***). Based on the mechanism of Fenton-like reaction and previous reports [5-7] an initial mineralization pathway has also been proposed (see **Fig. 6**). It can see that there are number conduits for mineralization of crystal violet dye as a function of interaction with oxidizing species such as hydroxyl free radicals, generated through Fenton reaction.

The removal of crystal violet dye was attributed to the synergistic effect caused by free radicals and other reactive species on the central carbon portion of crystal violet dye structure (see **Fig. 6-A**) [39]. As found in the results (discussed in **section 3.4**) upon oxidation, the dye becomes colourless, indicating the removal of the color bearing group and possible conversion into degraded intermediates. In the initial assumption, *R.E. Palma-Goyes et. al* (2010) [7] and *F. Guzman-Duque et. al* (2011) [8] suggested that due to oxidation crystal violet may degrade into 4-(N,N-dimethylamino)-4′-(N′,N′-dimethylamino) benzophenone (See **Fig. 6-B**) or 4-(N,N-dimethylamino)-4′-(N′,N′-dimethylamino) diphenylmethane (see **Fig. 6-C**).

However, a secondary degradation of these degraded intermediates occurs due to constant interaction with hydroxyl free radicals in the reaction bath and further degradation into 4-(N,N-dimethylamino)-4′-(N′,N′-dimethylamino) dimethylaniline (See **Fig. 6-D**) [6]. Finally, the gradual cleavage of the aromatic degraded intermediates would lead to mineralize into carboxylic acids prior to dissociate into H_20 and CO_2 as illustrated in ***Reaction 5-6***.

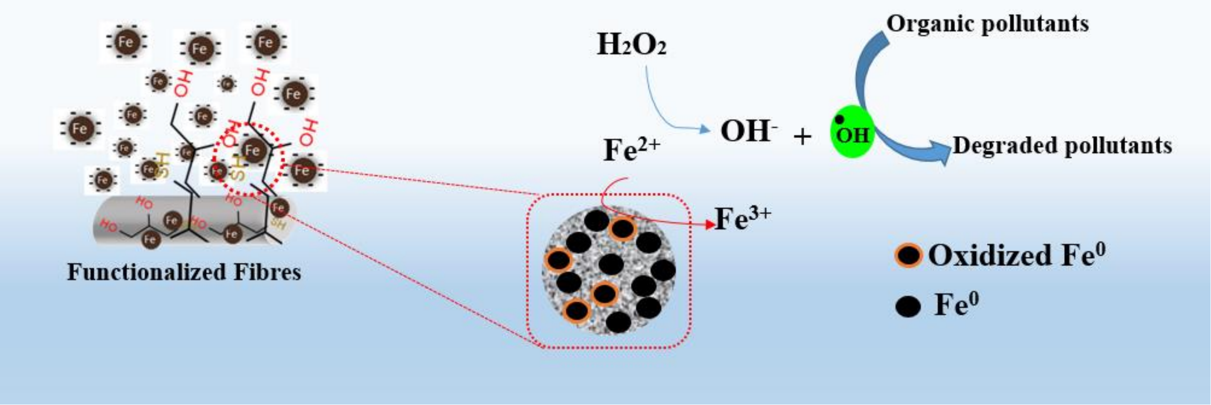

Fig. S5: Schematic postulated mechanism of removal of crystal violet dye as a potential organic pollutant. *[Same as: 10.1039/d0ra01362e]*

The postulates reaction mechanism involves three steps as follows;

(i) The process of producing iron ions and reactive species

$Fe^0 + 2H^+ \rightarrow Fe^{2+} + H_2$ (1)

$Fe^0 + 2Fe^{3+} \rightarrow 3Fe^{2+}$ (2)

$Fe^0 + H_2O_2 + 2H^+ \rightarrow Fe^{2+} + 2H_2O$ (3)

$Fe^{2+} + H_2O_2 \rightarrow Fe^{3+} + HO^{-} + \bullet OH$ (4)

(ii) The process of colour removal

Crystal violet + •OH → Degraded intermediates (5)

(iii) The process of mineralization

Degraded intermediates + •OH → $CO_2 + H_2O$ (6)

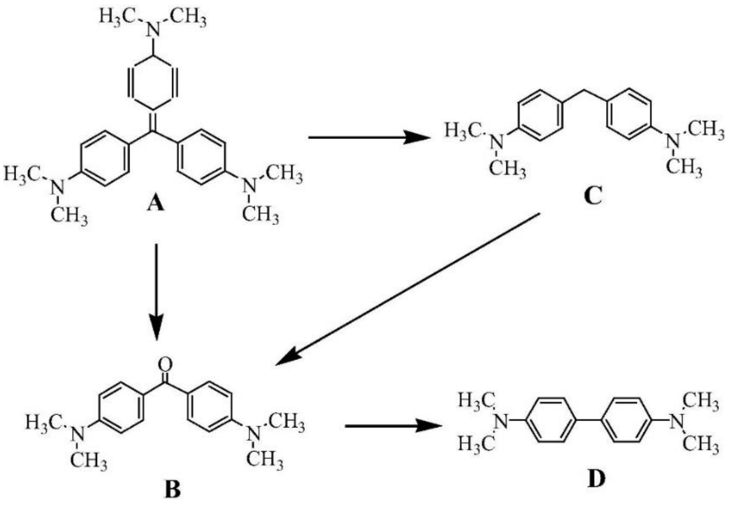

Fig. S6: Initial mineralization pathway of crystal violet dye *[Same as: 10.1039/d0ra01362e]*

References

[1] V. Takke, N. Behary, A. Perwuelz, C. Campagne, Studies on the atmospheric air–plasma treatment of PET (polyethylene terephtalate) woven fabrics: effect of process parameters and of aging, Journal of applied polymer science, 114 (2009) 348-357.

[2] M.N. Morshed, N. Bouazizi, N. Behary, J. Vieillard, O. Thoumire, A. Azzouz, Iron-loaded amine/thiol functionalized polyester fibers with high catalytic activities: Comparative study, Dalton Transactions, 48 (2019) 8384-8399.

[3] M.N. Morshed, N. Bouazizi, N. Behary, J. Vieillard, O. Thoumire, V. Nierstrasz, A. Azzouz, Iron-loaded amine/thiol functionalized polyester fibers with high catalytic activities: a comparative study, Dalton Transactions, 48 (2019) 8384-8399.

[4] A. Mohamed, B. Nemeshwaree, M. Brigitte, P. Anne, B. Kalim, D. Pascal, M. Anne-Sophie, F. Rénato, Activity of enzymes immobilized on plasma treated polyester, Journal of Molecular Catalysis B: Enzymatic, 134 (2016) 261-272.

[5] X. Wang, Y. Gao, W. Wang, A. Qin, J.Z. Sun, B.Z. Tang, Different amine-functionalized poly (diphenylsubstituted acetylenes) from the same precursor, Polymer Chemistry, 7 (2016) 5312-5321.

[6] R.E. Palma-Goyes, F.L. Guzmán-Duque, G. Peñuela, I. González, J.L. Nava, R.A. Torres-Palma, Electrochemical degradation of crystal violet with BDD electrodes: Effect of electrochemical parameters and identification of organic by-products, Chemosphere, 81 (2010) 26-32.

[7] H. Zhang, J. Wu, Z. Wang, D. Zhang, Electrochemical oxidation of Crystal Violet in the presence of hydrogen peroxide, Journal of Chemical Technology & Biotechnology, 85 (2010) 1436-1444.

[8] F. Guzman-Duque, C. Pétrier, C. Pulgarin, G. Peñuela, R.A. Torres-Palma, Effects of sonochemical parameters and inorganic ions during the sonochemical degradation of crystal violet in water, Ultrasonics sonochemistry, 18 (2011) 440-446.
